# How ‘smart’ is smart dentistry?

**DOI:** 10.12688/f1000research.17972.2

**Published:** 2019-07-16

**Authors:** Peter Kokol, Helena Blažun Vošner, Jernej Završnik, Marko Turčin

**Affiliations:** 1 Faculty of Electrical Engineering and Computer Science, University of Maribor, Maribor, 2000, Slovenia; 2 Community Healthcare Center Dr. Adolf Drolc, Maribor, 2000, Slovenia; 3 Faculty of Health and Social Sciences Slovenj Gradec, Slovenj Gradec, Slovenia

**Keywords:** smart medicine, smart dentistry, bibliometric mapping, papers as subjects

## Abstract

**Background: **Latest advances in information and health technologies enabled dentistry to follow the paradigm shift occurring in medicine – the transition to so called smart medicine. Consequently, the aim of this paper is to assess how ‘smart’ is smart dentistry as of the end of 2018.

**Methods: **We analysed the state of the art in smart dentistry, performing bibliometric mapping on a corpus of smart dentistry papers found in the Scopus bibliographical database.

**Results:** The search resulted in a corpus of 3451 papers, revealing that smart dentistry research is following the progress in smart medicine; however, there are some gaps in some specific areas like gamification and use of holistic smart dentistry systems.

**Conclusions:** Smart dentistry is smart; however, it must become smarter.

## Introduction

Advances in information, communication and health technologies triggered a paradigm shift in modern medicine – the transition to so called smart medicine. Some of the first appearances of the term smart medicine in the above context appeared in the late eighties and ninetieths in relation to (1) smart medical systems in the Space Station ^1^, (2) nuclear medicine and surgery ^2^ and (3) advanced biomimetic materials ^3^. In the beginning of the third millennia the research literature production on this subject started to grow. New smart application were introduced, like robotics surgery ^4^, smart medical systems in nutrition ^5^, smart medical records ^6^ and smart sensors ^7^. Recently, additional new smart health technologies including personalized and precision medicine, gamification based treatment, artificial intelligence, 3D printing, nanotechnology, Internet of Things and semantic health records have emerged ^8– 10^. Recently, dentistry started to follow smart medicine trends ^11^ and the aim of this paper is to assess the ‘smartness’ of smart density using a bibliometric approach. Due to the lack of “gold standards” it is not yet clear what may be considered *smart* or *not smart* technology in medicine or dentistry. Thus, in the absence of better metrics, we assessed the dentistry “smartness” with the frequency of use of the above listed smart technologies.

## Methods

To analyse the state of the art in smart dentistry, we analysed the corpus of papers retrieved from the Scopus bibliographical database (Elsevier, Netherlands). The search string was composed from representative keywords found in smart medicine research in the following manner: *smart* or *personalized* or *precision or G4H* or *“artificial intelligence”* or “3D print*” or *nanotechnology* or robotic* or *IoT* or *“semantic health record”*. The search was restricted to the subject area of dentistry (which in Scopus includes dentistry, endodontics, oral health, oral biology, orthodontics, prosthodontics and periodontology). We limited the search to the period beginning in 2001, when the growth of literature production on smart medicine began, up to 2018 (inclusive) and articles published in journals only. Using descriptive bibliometrics we identified the research literature production trends, most productive countries and most prolific journals.

To analyse and visualize the context of the smart density research literature we used a bibliometric mapping approach and a popular mapping tool called VOSViewer Version 1.6.9 (Leiden University, Netherlands) ^12, 13^. The outputs from VOSViewer are various types of bibliometric maps, frequently called science landscapes. Landscapes can reveal different patterns and aspects of research literature like associated or related terms/keywords, timelines, citation, country or networks and similar. In our study, the author cluster keyword landscape was induced using “Create a map based on bibliographic data” option in the opening VOSViewer menu. After selecting Scopus as the bibliographic database used and defining the names of files to be analysed we selected “Co-occurrence Author Keywords” as the type of analysis and “Full counting” as the counting method. Then we set the “Minimum number of occurrences of a keyword” to 8 occurrences. For all other parameters the default values were used. The proximity of terms indicates keyword similarity and the coloured clusters represent strongly associated keywords. Using a customized VOSViewer thesaurus file, we excluded common and statistical keywords like *systematic review or meta-analysis* from the analysis. We also mapped synonyms into one entity (for example *cone beam computer tomography, cone-beam computer tomography, cone beam computed tomography, cone-beam computed tomography* and *cbct* into *cone-beam computer tomography*). The thesaurus file is consisting of two columns, first includes the synonym and second the keyword in which the synonym should be mapped. To omit a keyword from the analysis, the second column entry is left empty.

## Results

The search was performed on 12 ^th^ of December 2018 and resulted in a corpus of 2470 papers. The research literature production exhibits the linear growing trend from 2001 till 2016, namely from 46 to 198 articles per year, with the average increase of nine papers per year. In last two years the growth was still linear, however with an average increase of 78 articles per year. The productivity reached its peak in 2018 with 353 articles.

The most productive countries were United Stated of America (USA) (n=627), Germany (n=298), Brazil (n=223), Italy (n=174), United Kingdom (UK) (n=168), India (n=1266), Japan (n=120), South Korea (n=111). Switzerland (n=110) and China (n=119). The top 10 productive countries are belonging either to the G8 group or are countries with highly developed economies and health systems. The most prolific journals are Journal of Prosthetic Dentistry (n=131), Dental Materials (n=83), Oral Oncology (n=79), Journal of Oral and Maxillofacial Surgery (n=75), Journal of Dental Research (n=71), American Journal of Orthodontics and Dentofacial Orthopaedics (n=59), Clinical Oral Implants Research (n=51) and Clinical Oral Investigation (n=49). Top journals belong to the most prestigious and highest-ranking journals in the dentistry field.

**Figure 1.**
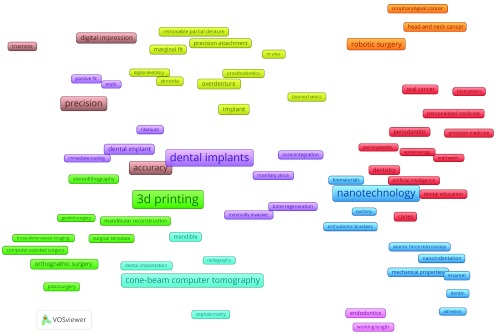
The author keywords cluster science landscape of smart dentistry research.

Nine clusters ( Figure 1) emerged on the cluster landscape. We used the cluster keywords as codes in the thematic analysis ^14^, focusing on “medical smartness”. In that manner the following smart dentistry themes were derived:

- **Digital impression** (brown colour): Digital impressions represent cutting-edge technology that allows dentists to create an accurate virtual, computer-generated replica of the hard and soft tissues in the mouth using advance 3D scanning devices in a very short time. In that manner, the use of traditional impression materials that some patients find inconvenient, can be avoided. Digital optical impressions significantly increase efficiency, productivity and accuracy, and enable dentists to distribute impressions using e-mails. Digital impressions in combination with 3D print can be used to make immediate restorations, reducing the need for patients multiple office visits ^15^.
- **Digital dentistry in prosthodontics (**yellow colour **).** As the name applies Digital density is focused on use of digital technologies in dentistry in general, but focusing on prosthodontics ^16, 17^, however, it also deals with smart management of patients ^18^.
- **Dental implants and computer aided design** (violet colour): The advance in dental materials required a new of design in dental practice. In that manner, computer aided design (CAD) has been introduced into dentistry ^19^. CAD is also used for the reconstruction of face defects due to flaps or bone defects ^20^.
- **Robotic surgery (orange colour)** is mainly used in transoral neck and head surgery ^21^. Especially interesting is the application of robotics removal of very rare parapharyngeal space tumours ^22^. On the other hand, computer assisted surgery is mostly used in mandibular reconstruction ^23^.
- **Biomaterials and nanotechnology in tissue engineering and endodontics** (blue and pink colours): The idea of biomaterials in dentistry is to have a dynamic’ smart behaviour in the manner that the materials can react to changes in the environment with the advantageous changes in their properties to benefit the dental patient. These smart materials can react to stress, temperature, moisture, pH, etc. A promising version of them are bio-smart materials ^24^. Smart materials include nanomaterials which are mainly used to fight caries, to enhance remineralization of apatite-depleted dentin, dental tissue regeneration and drug delivery ^25, 26^. On the other hand, smart brackets tend to be more efficient in reducing treatment times compared to conventional bracket, however, the quality of orthodontic treatment is similar to conventional systems as is the patient perception. An interesting recent upgrade in smart brackets is the integration of sensors, which can measure forces and moments used to improve treatment ^27^.
- **Artificial intelligence and precision/personal medicine in dentistry** (red colour): Recently, the artificial intelligence has been introduced in dentistry to achieve the goals of precision and personalised health care ^28^. It is used in decision making ^29^, evaluation of facial attractiveness with maloocclusion ^30^, diagnosing ^31^ and similar technologies ^32^.
- **3D printing in surgery, implantation and reconstruction** (green and light blue colour): 3D printing has many applications in dentistry and showed improvements in precision and reduction, surgery times and personalisation ^33^. In combination with cone beam commuted tomography ^34^ and CAD, 3D printing has been successfully used in various endodontic challenges ^35^.

From a quantitative point of view, the most prolific smart medicine technologies used in dentistry are 3D printing occurring in 99 articles, nanotechnology occurring in 80 articles, robotic surgery occurring in 43 articles, digital impression occurring in 33 articles and artificial intelligence occurring in 13 articles.

## Discussion

The above analysis showed that smart dentistry in general is following smart medicine “movement” especially in using 3D printing, nanotechnology and smart materials, robotics, IoT (i.e. sensors) technologies, personalised and precision medicine and artificial intelligence. The three examples of technologies/approaches which brought most smartness to the dentistry are smart materials, which can be altered in a controlled manner by pH, various fields, temperature, stress, etc to mimic for instance enamel and dentin or execute desired intelligent functions like diagnostic and regeneration; real time imaging and CAD/CAM systems setting foundations for increase in precision in robotic surgery; and artificial intelligence which aids quick diagnosis and customises treatment planning based on myriad of data gathered from 3D scans, cone beam computed tomography and similar complex devices.

## Conclusion

Despite many applications of smart technologies in dentistry, there are still substantial gaps. Smart medicine technologies regarding gamification, deep-learning or semantic eHealth dentistry records are yet to be started to use. Thus, in absence of “gold standards” we may state that the smart dentistry is smart, but to be really successful it must become smarter.

## Data availability

### Underlying data

OSF: Dataset 1. Smart dentistry. https://doi.org/10.17605/OSF.IO/UJRKT ^36^

Licence: CC0 1.0 Universal
