# Supplementary material for: How ‘smart’ is smart dentistry?
Source: F1000Res. 2019 Jul 16;8:183. Originally published 2019 Feb 14. [Version 2] doi: 10.12688/f1000research.17972.2 (PMC6696613; doi:10.12688/f1000research.17972.2)
Supplement: Peer review history

# Peer review: Reviewer response for version 2

**DOI:** 10.5256/f1000research.21815.r51267

**Published:** 2019-08-15

**Authors:** Praveen Arany

**Affiliations:** 1 Department of Oral Biology, School of Dental Medicine, University at Buffalo, Buffalo, NY, USA; 2 Department of Biomedical Engineering, School of Engineering and Applied Sciences, University at Buffalo, Buffalo, NY, USA

The authors have largely addressed concerns raised. This is a great effort to showcase this exciting new frontier for dentistry - Congratulations!

I confirm that I have read this submission and believe that I have an appropriate level of expertise to confirm that it is of an acceptable scientific standard.

# Peer review: Reviewer response for version 1

**DOI:** 10.5256/f1000research.19655.r49289

**Published:** 2019-07-01

**Authors:** Praveen Arany

**Affiliations:** 1 Department of Oral Biology, School of Dental Medicine, University at Buffalo, Buffalo, NY, USA; 2 Department of Biomedical Engineering, School of Engineering and Applied Sciences, University at Buffalo, Buffalo, NY, USA

The advances of biomedical technologies and biomaterials is bringing about staggering improvements in clinical care, especially in dentistry that has always been at the forefront of adapting and utilizing the latest scientific advances. The authors do a good job of compiling the literature on this topic. This is a timely article as there is much excitement of how the rapid pace of technological advancements that are improving routine patient care.

 However, there seems to be fundamental confusion on what exactly constitutes a ‘Smart’ approach or technology. While digital impression, robotic surgery or precision additive (3D printing) manufacturing are phenomenal advances that are enabling individualized care, the techniques by themselves are not, in my personal opinion, a smart approach.

 The following are examples of what may not be considered ‘Smart’ based on their ability to simply perform an enhanced passive or active function. 

- Precision-designed implant surface is NOT considered smart because it is passively driving a directed osseointegration response;
- Drug eluting biomaterial is NOT considered smart despite actively releasing an antimicrobial or disease modifying agent. 

The classical definition, I believe, of a ‘Smart’ approach is a measured, controlled response to modulate a biological response based on some input/sensing. Some examples of Smart approaches are:

- pH or enzyme activated payload release from biomaterials;
- Sensors on appliances that enable (hardware/biomaterial) improved compliance or dose/treatment modulation;
- Big data (clinical, laboratory, epidemiological) and artificial intelligence enabling (software) improved precision and personalized care; 

Of the key topics/focus areas identified, the ‘smart’ aspect of each area could be specifically highlighted. For example, robotic surgery is a major technological advance but the use of diagnostic approaches, such as real-time radiographic or ultrasound imaging has enabled increased precision. Another key example in dentistry is the use of protease-sensitive dressings or materials that indicate active tissue turnover providing a visual cue for a clinical intervention.

I confirm that I have read this submission and believe that I have an appropriate level of expertise to confirm that it is of an acceptable scientific standard, however I have significant reservations, as outlined above.

# Peer review: Reviewer response for version 1

**DOI:** 10.5256/f1000research.19655.r47891

**Published:** 2019-05-29

**Authors:** Wan Zaripah Wan Bakar, Huwaina Abd Ghani

**Affiliations:** 1 Department of Prosthodontics and Biomaterials, School of Dental Sciences, Universiti Sains Malaysia, Kubang Kerian, Malaysia; 2 School of Dental Sciences, Universiti Sains Malaysia, Kubang Kerian, Malaysia

This article entitled “How ‘smart’ is smart dentistry?” is an interesting topic. It looks upon smart dentistry which is an important area especially as we are currently in the Fourth Industrial Revolution, where everything is changing fast with new technologies. It is no doubt that smart dentistry already exists with more new advanced technology that has emerged to facilitate clinical works.

 However with this bibliometric mapping methods study, the included papers were not critically appraised and the details information were not described clearly. Limitations that band it from being smarter such as cost and resources should be elaborated. It just show the overall general trend where a proper conclusion cannot be made, which ends up with very a weak conclusion. Maybe it is better to conclude that there is an improvement in smart dentistry with year changes especially in developed country as the gold standard level is yet not known.

We confirm that we have read this submission and believe that we have an appropriate level of expertise to confirm that it is of an acceptable scientific standard.
